# Validation of Peripheral Neuromodulation Mechanisms of Icariin in Knee Osteoarthritis–Related Chronic Pain

**DOI:** 10.1111/jcmm.70223

**Published:** 2024-12-02

**Authors:** Dezun Ma, Zaishi Zhu, Xue Tan, Qing Lin, Yanfeng Huang, Min Mao, Zhouping Yi, Lili Wang, Linglong Liu, Xihai Li

**Affiliations:** ^1^ Academy of Integrative Medicine Fujian University of Traditional Chinese Medicine Fuzhou Fujian China; ^2^ Fujian Key Laboratory of Integrative Medicine on Geriatrics Fuzhou Fujian China; ^3^ Suzhou TCM Hospital Affiliated to Nanjing University of Chinese Medicine Suzhou Jiangsu China; ^4^ College of Integrative Medicine Fujian University of Traditional Chinese Medicine Fuzhou Fujian China

**Keywords:** dorsal root ganglion, icariin, knee osteoarthritis, pain, subchondral bone

## Abstract

Knee osteoarthritis (KOA) is a chronic degenerative joint disease‐causing chronic pain and disability. Neuromodulation of subchondral bone affects KOA‐related pain and involves dorsal root ganglion (DRG). Our previous studies have demonstrated efficacy of icariin (ICA) in treating KOA, but neuromodulation mechanisms in peripheral nerves associated with the treatment of chronic pain in KOA remain unclear. This study aimed to investigate peripheral neuromodulation mechanisms of ICA in KOA‐related chronic pain: (1) assessing therapeutic effect of ICA in a rat model of KOA‐induced chronic pain; (2) investigating changes in pain‐related nerve fibres and transient receptor potential vanilloid Subfamily 1 (TRPV1) pathway in subchondral bone following ICA treatment; and (3) exploring expression of pain‐related Nogo‐A/TRPV1 pathway in DRG, thereby elucidating neurotransmission of pain. Experimental results confirmed the curative effect of ICA on KOA by relieving chronic pain and pathological changes. ICA also effectively reduced bone remodelling, the area of pain‐related positive nerve fibres and expression of TRPV1 in subchondral bone. Furthermore, ICA downregulated pain‐related Nogo‐A/TRPV1 pathway in the DRG. These findings provide new mechanistic insights into the therapeutic potential of ICA in relieving peripheral nervous system‐related chronic pain in KOA.

## 1 Introduction

Osteoarthritis (OA) is a chronic degenerative joint disease that is prevalent among the elderly and is characterised by articular cartilage degeneration and subchondral bone remodelling [1, 2]. It has emerged as one of the four major disabling diseases, ranking first among the global causes of disability and chronic pain [3]. Pain is an important indicator of the progression of OA, significantly impacting the quality of life for patients and indirectly contributing to cardiovascular problems and an increased mortality rate [4]. Therefore, the search for effective methods to relieve OA‐related pain is an urgent medical imperative.

Subchondral bone, unlike cartilage, contains nerves and blood vessels [5] and is involved in the pathogenesis of KOA‐related pain [6]. It possesses a dense network of sensory nerve endings that are responsive to noxious stimuli and can be sensitised by inflammation, which is closely associated with the pathophysiology of KOA‐related pain [7]. The knee joint is innervated by various types of sensory nerves, including fast‐conducting myelinated Aδ fibres, slow‐conducting unmyelinated Aβ fibres, thick myelinated Aβ fibres and sympathetic nerve endings. The afferent neurons of the subchondral bone are predominantly immunoreactive to calcitonin gene‐related peptide (CGRP), tyrosine receptor kinase A (TrkA) and neurofilament 200 (NF200) [8]. The peripheral nerve fibres of the subchondral bone can be traced back to the DRG. The cell bodies of the primary sensory neurons innervating the subchondral bone are located in the DRG. These neurons can be classified into two groups based on their neurochemical properties: one group contains neuropeptides such as CGRP and substance P (SP), while the other group lacks neuropeptides but binds to isolectin B4 [9].

TRPV1, as a nociceptive nonselective cation channel, plays an important role in detecting noxious stimuli and inflammatory hyperalgesia. It is enriched in the small‐diameter cell bodies of nociceptive nerve fibres innervating joints and joint capsules, and its expression is upregulated in sensory afferent fibres innervating OA joints [10, 11]. TRPV1 is associated with pain in both animal models of OA and human patients with OA [12]. In addition, TRPV1 is involved in the formation of osteoblasts and osteoclasts in the subchondral bone and contributes to bone pain, and its blockers have been shown to relieve OA‐related pain. The efficacy of axon terminal ablation therapy targeting TRPV1 channels in neuropathic pain has been clearly demonstrated [13]. Nogo‐A, encoded by the *RTN4* gene, is a mammalian axon growth regulator that is widely distributed in both the central nervous system (CNS) and peripheral nerve fibres [14]. It functions to inhibit axon regeneration and regulate the structure and function of the endoplasmic reticulum during CNS injuries [15]. Nogo‐A can also relieve inflammation‐induced pain mediated by the peripheral nervous system by inhibiting the activation of TRPV1 channels [16]. In inflammatory pain, Nogo‐A maintains the functionality of TRPV1 by activating the LIM domain kinase/cofilin pathway through microtubule polymerisation. The use of a Nogo‐A receptor antagonist peptide has been shown to effectively improve inflammatory thermal hyperalgesia by reducing the expression of TRPV1 in the DRG [17].

Epimedium is a commonly used kidney‐tonifying drug in traditional Chinese medicine. Its main active component, ICA, has been found to have multiple therapeutic effects on OA [18]. ICA can promote chondrocyte proliferation and chondrogenic differentiation of bone marrow mesenchymal stem cells [19]. It also improves OA cartilage degeneration and subchondral and abnormal bone remodelling of osteoblasts and osteoclasts [20, 21]. Additionally, ICA has been shown to effectively relieve OA‐related pain [22]. Computer molecular docking simulations have revealed that ICA binds well to the TRPV1 receptor, suggesting that ICA may relieve neuropathic pain through the TRPV1 pathway [23]. Furthermore, the ability of ICA to relieve OA‐related pain is believed to be related to its neuromodulatory effects. Nogo‐A, for instance, can inhibit TRPV1 channel activation to relieve pain [24]. However, the specific neural mechanism by which ICA can relieve KOA‐related pain remains unclear.

This study aimed to investigate the effect of ICA on pain in KOA. Three verifications were carried out, as follows: (1) The efficacy of ICA and its effect on KOA‐related pain were verified in traditional KOA animal models using pain‐related indicators, including paw withdrawal threshold (PWT) and paw withdrawal latency (PWL); (2) the therapeutic effect of ICA on subchondral bone remodelling and TRPV1 expression in peripheral nerve fibres was verified based on the results of Western blot and immunofluorescence analyses of subchondral bone; and (3) the relationship between ICA's relief of KOA‐related pain and the Nogo‐A/TRPV1 pathway was verified based on the results of Western blot and immunofluorescence analyses in DRG. Accordingly, it was confirmed that ICA plays a role in relieving pain associated with peripheral nerve effects in KOA. These findings contribute to the development of ICA as a novel therapy for KOA and provide evidence of its association with pain.

## 2 Experimental Procedures

### 2.1 Animals

Male Sprague–Dawley rats weighing 200–220 g were obtained from the Animal Center of the Fujian University of Traditional Chinese Medicine. The rats were bred and housed in the Experimental Animal Center of Fujian University of Traditional Chinese Medicine under licence number (Fujian): SYXK2019‐0007. The raising conditions included a room temperature of 22°C–26°C and a relative humidity of 40%–70%. They were treated according to the National Institutes of Health Guidelines for the Care and Use of Laboratory Animals. All experiments were approved and supervised by the Animal Care and Use Committee of the Fujian University of Traditional Chinese Medicine. This study was reviewed and approved by the Animal Care and Use Committee of the Fujian University of TCM (Approval Number: 2019061).

### 2.2 KOA Rat Model

Thirty rats were randomly divided into three groups: the sham group (*n* = 10); the KOA group (*n* = 10); and the ICA group (*n* = 10). The KOA model was established using a modified Hulth method, as described in our previous study [25]. Briefly, after anaesthesia with isoflurane (1.5%–2%), the animals underwent surgery. In 20 rats, the medial meniscus was resected. In the sham group, rats were only exposed to the knee joint cavity, and the incisions were sutured without any additional procedures. Herein, 2 weeks after the operation, the rats were randomly selected for ICA treatment (20 mg/kg, once/day, i.g.).

### 2.3 Pain‐Related Behaviour Assessment

According to previous studies [26], PWT and PWL were used as pain‐related indicators. Briefly, PWT was measured using a digital force gauge (YLS‐3E, Yima Optoelectronics Co. Ltd., China). A rising force was applied to the hind paw until a flinch reflex occurred. Animals were subjected to PWL measurement using a thermal pain stimulator (BME‐410C, Biomedical Engineering Institute, USA) and a 12 V/10 W halogen lamp. Three measurements were performed for each animal, and the average value was recorded.

### 2.4 Microcomputed Tomography Scans

Changes in the knee joint were observed using micro‐CT. The scanning parameters were set as a scanning tube voltage of 50 kV, a current of 100 μA, a scanning time of 4 min and complete scanning with one rotation.

### 2.5 Haematoxylin and Eosin, Safranin O/Fast Green and Masson's Staining

The resected tibial plateaus were fixed in 4% paraformaldehyde for 48 h. After decalcification with 10% ethylenediaminetetraacetic acid disodium salt for 8 weeks, the tissue blocks were embedded in paraffin. The embedded tissue sections were routinely deparaffinised and stained with haematoxylin and eosin (H&E), Safranin O/fast green (SO/FG) and Masson's stains (Sorabio, China) in sequence, following the instructions provided with the staining kit. The morphological structure was examined using a digital microscope (IX‐71, Olympus, Japan). The modified Wakitani scoring system was used to score rat tissue sections [27].

### 2.6 Immunofluorescence Detection

The localisation and content changes of TRPV1 (1:100, Abcam, USA), CGRP (1:200, Cell Signalling Technology, USA), TrkA (1:100, R&D Systems, USA), NF200 (1:500, Abcam) and vascular endothelial growth factor (VEGF; 1:100, sc‐7269, USA) in the subchondral bone were detected using the immunofluorescence method. ImageJ was used to detect the intensity and area of the corresponding fluorescence, and we counted the number of colocalisations.

### 2.7 Western Blot Analysis

Standard Western blot analysis was performed to detect the protein expression of TRPV1 (1:1000, Abcam), nerve growth factor (NGF; 1:1000, Abcam) and subchondral bone remodelling‐related proteins alkaline phosphatase (ALP; 1:1000, Invitrogen, USA), tartrate‐resistant acid phosphatase (TRAP; 1:1000, Abcam), osteoprotegerin (OPG; 1:1000, Abcam) and receptor activator of nuclear factor‐kappa B ligand (RANKL; 1:1000, Proteintech, China) in the subchondral bone. Additionally, the protein expression of TRPV1 (1:1000, Abcam) and Nogo‐A (1:1000, Invitrogen) in the DRG was examined. Vinculin (1:1000, Proteintech) and β‐actin (1:1000, Abcam) were used as the reference protein.

### 2.8 Enzyme‐Linked Immunosorbent Assay (ELISA)

The animals were anaesthetised with isoflurane (1.5%–2%), and blood was collected from the vena cava. The collected blood was centrifuged at 10,000 rpm for 30 min at 4°C. The upper serum layer was used for ELISA. Levels of neuropeptides SP, tyrosine hydroxylase (TH) and CGRP were measured using ELISA kits (BioTek, USA; Cloud‐Clone Corp., China) following the instructions of the manufacturer.

### 2.9 Data Analysis

The research results were statistically processed using the Statistical Package for the Social Sciences (SPSS) 25.0 software, and GraphPad Prism 8.0 software was used for statistical graph processing.

## 3 Results

### 3.1 Validation of the Therapeutic Effects of ICA

**FIGURE 1:**
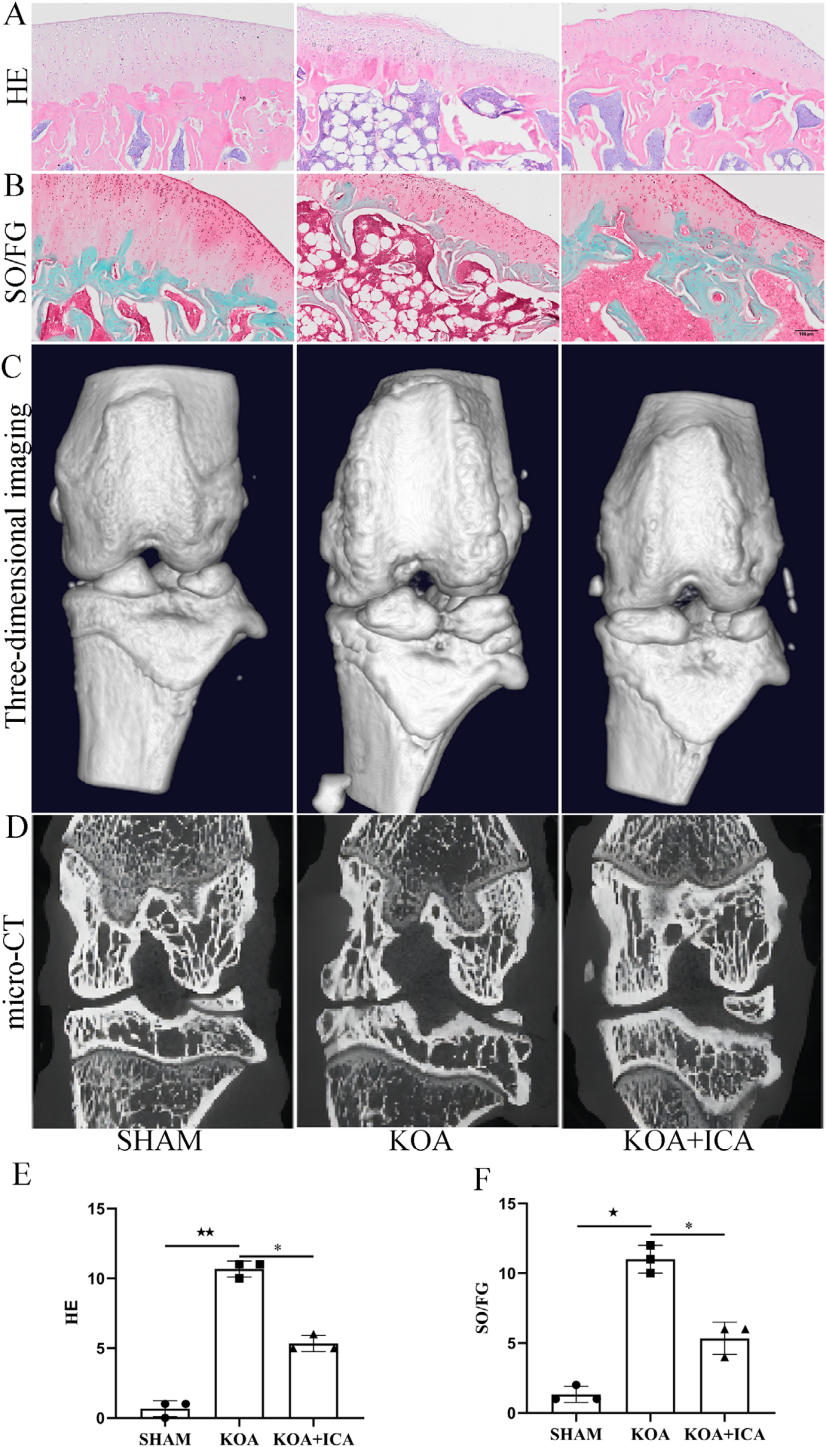
Evidence of the efficacy of ICA therapy. Representative images of HE (A) and SO/FG staining (B) are presented (scale bar = 100 μm). In KOA, degeneration of the cartilage surface is observed, with visible collagenous fibres on the articular surfaces of both the femur and tibia. ICA treatment appears to alleviate these pathological changes, resulting in smoother cartilage surfaces. Representative microcomputed tomography scan images (C, D) provide additional evidence supporting these findings. (E, F) Histological scoring of cartilage repair using the modified Wakitani score. ^★^ *p* < 0.05, ^★★^ *p* < 0.01, *p < 0.05. ^★^Comparison between SHAM and KOA, *comparison between KOA and KOA + ICA.

In KOA, the cartilage layer became thinner, with distorted chondrocytes, an uneven distribution of the cartilage matrix, the formation of a large number of cell clusters and a noticeable loss of proteoglycan. However, these pathological changes improved after ICA intervention compared to the KOA group (Figure 1A,B). These findings were further confirmed by micro‐CT scans. In the KOA group, osteophytes formed around the joint, and the trabecular bone showed sparsity, decreased thickness, number and continuity. The arrangement of trabecular bone became irregular, with widened spacing and the formation of some cavities. In comparison, the ICA group exhibited fewer osteophytes, thicker and denser trabeculae, a more uniform arrangement, better bone continuity, more trabeculae, denser distribution and a more orderly texture, indicating a significant improvement in bone microstructure (Figure 1C,D). The results of the modified Wakitani score showed that the model group had higher scores than the control group. In addition, the scores decreased after ICA intervention (Figure 1E,F). Therefore, we have successfully established a rat model of KOA and demonstrated the therapeutic effect of ICA. Furthermore, it was observed that KOA led to a significant decrease in pain threshold, which could be reversed by ICA, although not completely.

### 3.2 Validation of the Curative Effect of ICA on KOA‐Related Pain

**FIGURE 2:**
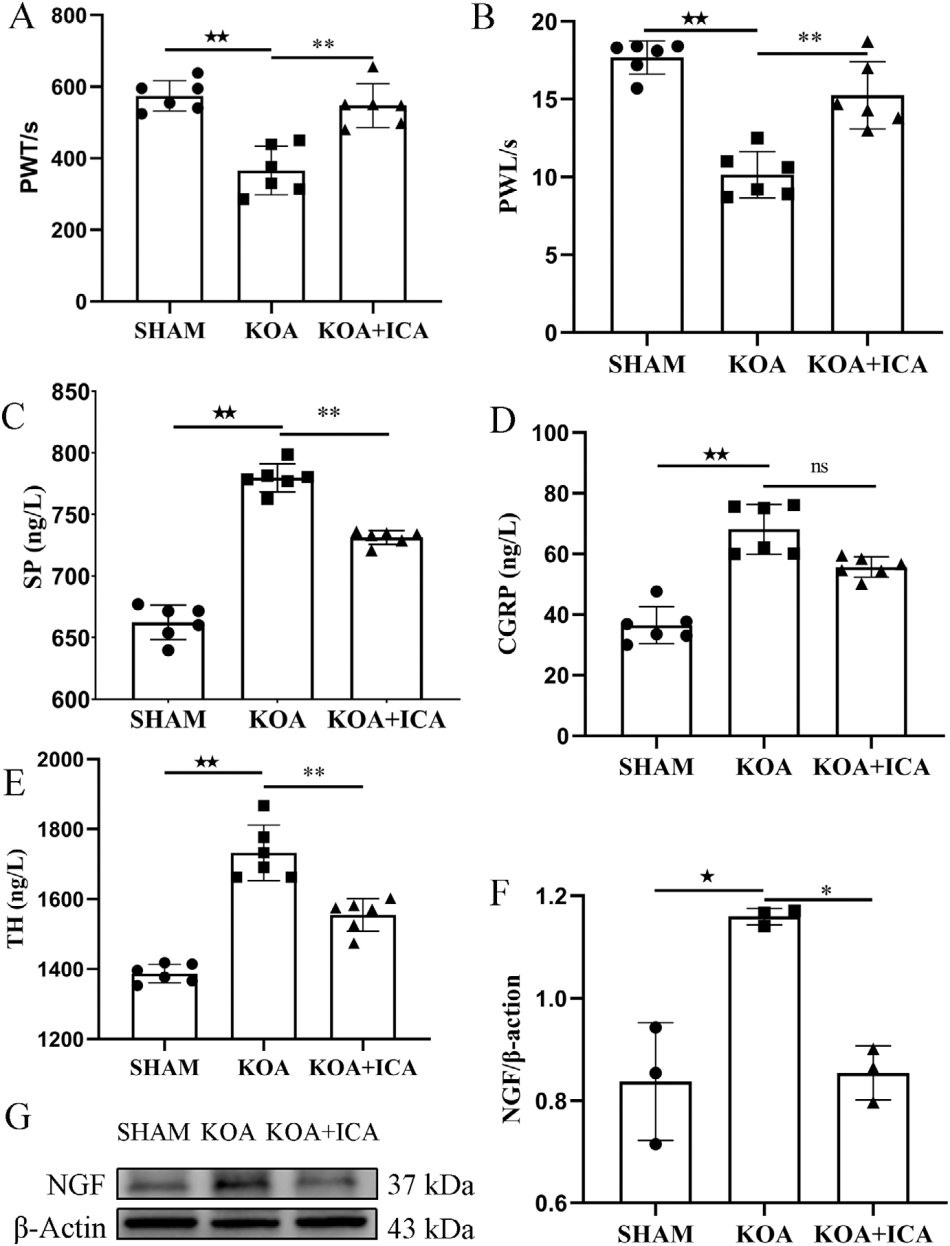
ICA improves pain in KOA rats. PWT and PWL were used to assess pain. KOA significantly reduced the PWT and PWL, whereas ICA treatment significantly increased the PWT and PWL of rats (A, B). The serum levels of SP, TH and CGRP in KOA increased (*p* < 0.01), while those of SP, TH (*p* < 0.01) and CGRP (*p* > 0.05) decreased upon ICA treatment (C–E). Protein expression level of NGF by Western blot in the subchondral bone (F, G). *p < 0.05, **p < 0.01, ^★^ *p* < 0.05, ^★★^ *p* < 0.01, ns for no significance. *Comparison between SHAM and KOA, ^★^comparison between KOA and KOA + ICA.

PWL and PWT were significantly decreased in the KOA group, as indicated by pain behaviour, and were significantly increased after ICA intervention (Figure 2A,B). Our morphological experiments confirmed the pathological characteristics of KOA and demonstrated the therapeutic effect of ICA intervention.

Neuropeptides, including CGRP, SP, TH and others, are small peptide compounds present in the nervous system and many other tissues. In the context of KOA‐related pain, neuropeptides such as CGRP, SP and TH are involved in the transmission and regulation of pain signals. SP, in particular, is considered an important regulator of KOA‐related pain. It can activate neurons and inflammatory cells, contributing to pain perception and the exacerbation of the inflammatory response [28]. The SP levels in the serum of patients with OA have been reported to significantly increase and positively correlate with the severity of pain. CGRP, another pain‐related neuropeptide, plays an important role in the transmission and regulation of pain sensation in OA and can synergise the inflammatory and pain‐inducing effects of SP. In patients with OA, the levels of CGRP have been reported to significantly increase in the articular cartilage and synovial fluid. CGRP can contribute to pain sensation and the exacerbation of the inflammatory response by activating neurons and inflammatory cells. TH can interact with other pain regulators, such as CGRP, SP and others, to participate in the occurrence and maintenance of OA‐related pain. However, ICA can inhibit pain signal transmission by regulating the expression of serum SP, CGRP and TH neuropeptides (Figure 2C–E).

NGF is involved in the process of pain perception and transmission in OA. In the pathological changes associated with KOA, NGF levels and expressions have been reported to significantly increase in close association with the generation of pain [29]. In this study, ICA was found to reduce the expression of NGF in the subchondral bone of KOA rats (Figure 2F,G).

### 3.3 ICA Improves Bone Remodelling by Improving the Distribution of Positive Nerve Fibres in the Subchondral Bone

**FIGURE 3:**
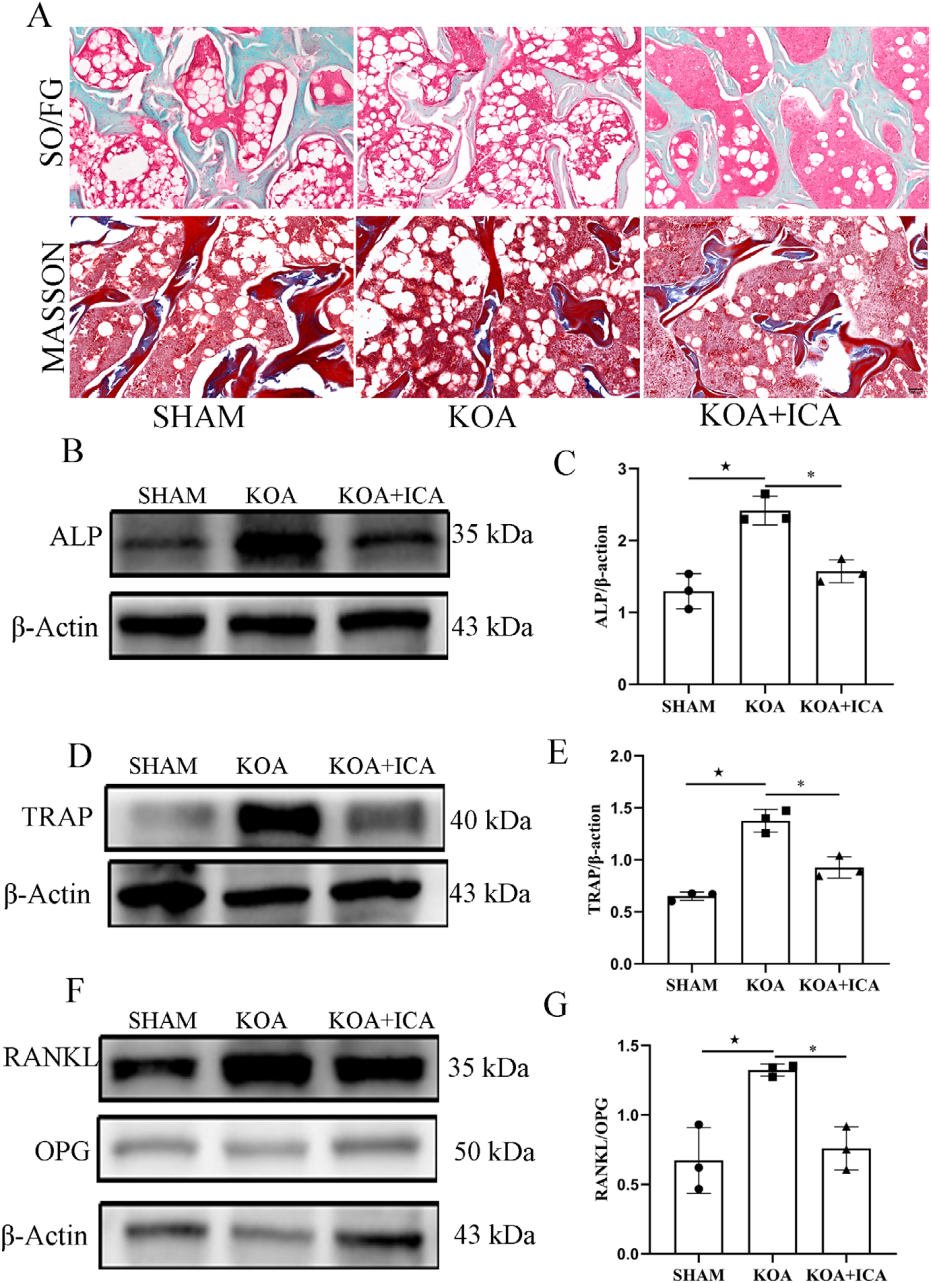
ICA improves subchondral bone remodelling in KOA. Representative images of the subchondral bone stained with SO/FG and Masson's staining (A) (scale bar = 100 μm). Representative images and quantitative analysis of bone remodelling indexes in the subchondral bone (B–G). *p < 0.05, ^★^ *p* < 0.05. ^★^Comparison between SHAM and KOA, *comparison between KOA and KOA + ICA.

The imbalance of bone resorption and formation homeostasis is an important pathological feature of KOA. Through morphological observation of the subchondral bone, it was found that the trabecular bone became thin and sparse, and new bone appeared in the KOA group. However, after ICA intervention, the trabecular bone became thicker and denser, and its continuity and integrity improved (Figure 3A). The biomarkers of bone remodelling in the subchondral bone showed a similar trend, with ALP, TRAP and RANKL/OPG upregulated in the KOA group and significantly inhibited by ICA (Figure 3B–G).

**FIGURE 4:**
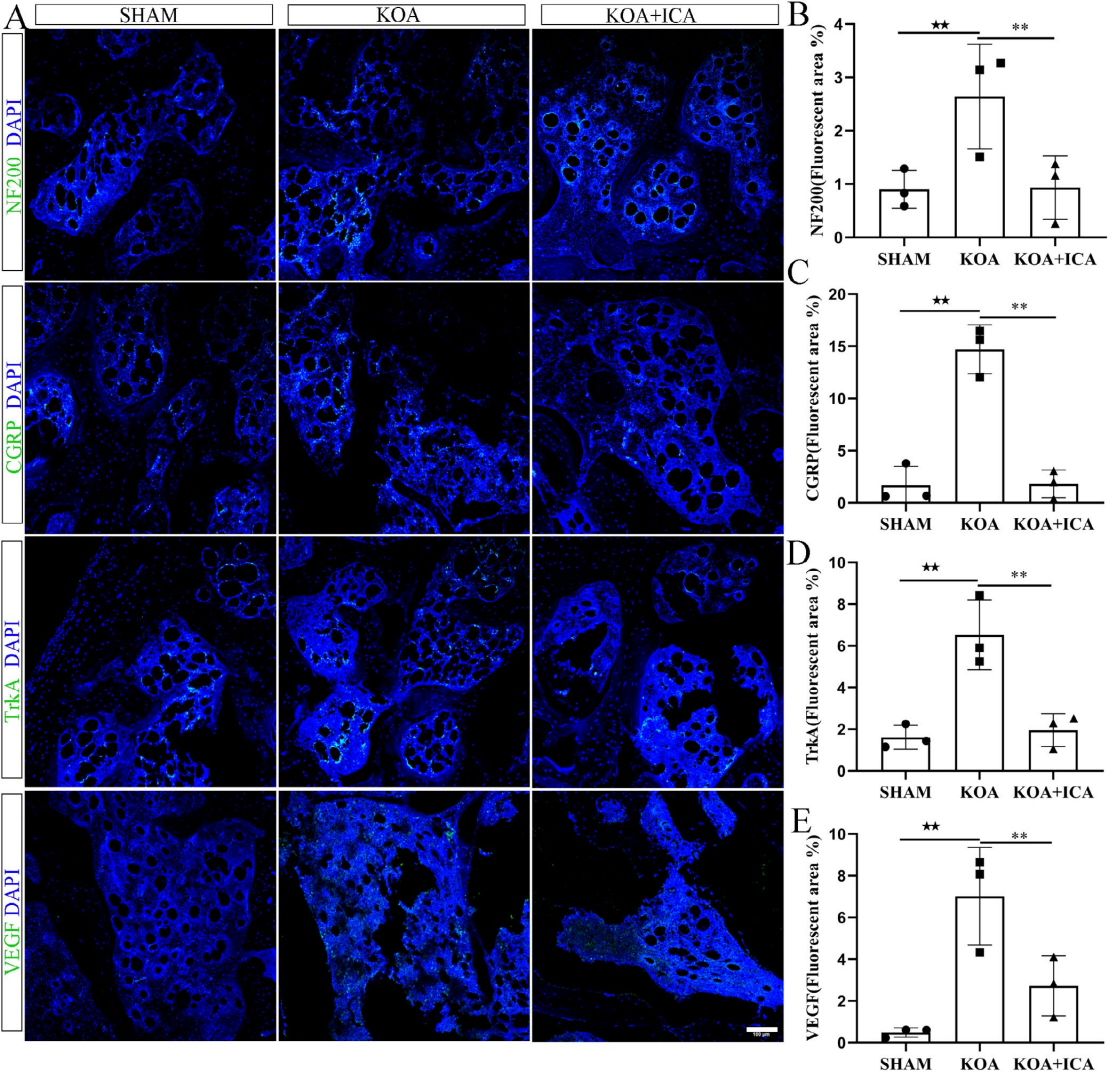
ICA inhibits the expression of positive nerve fibres in the subchondral bone. Representative immunofluorescence images of subchondral bone nerve fibre CGRP, TrkA, NF200 and VEGF, along with quantitative analysis of the positive fibre area (A–E) (scale bar = 100 μm). ^★★^ *p* < 0.01, **p < 0.01. ^★^Comparison between SHAM and KOA, *comparison between KOA and KOA + ICA.

**FIGURE 5:**
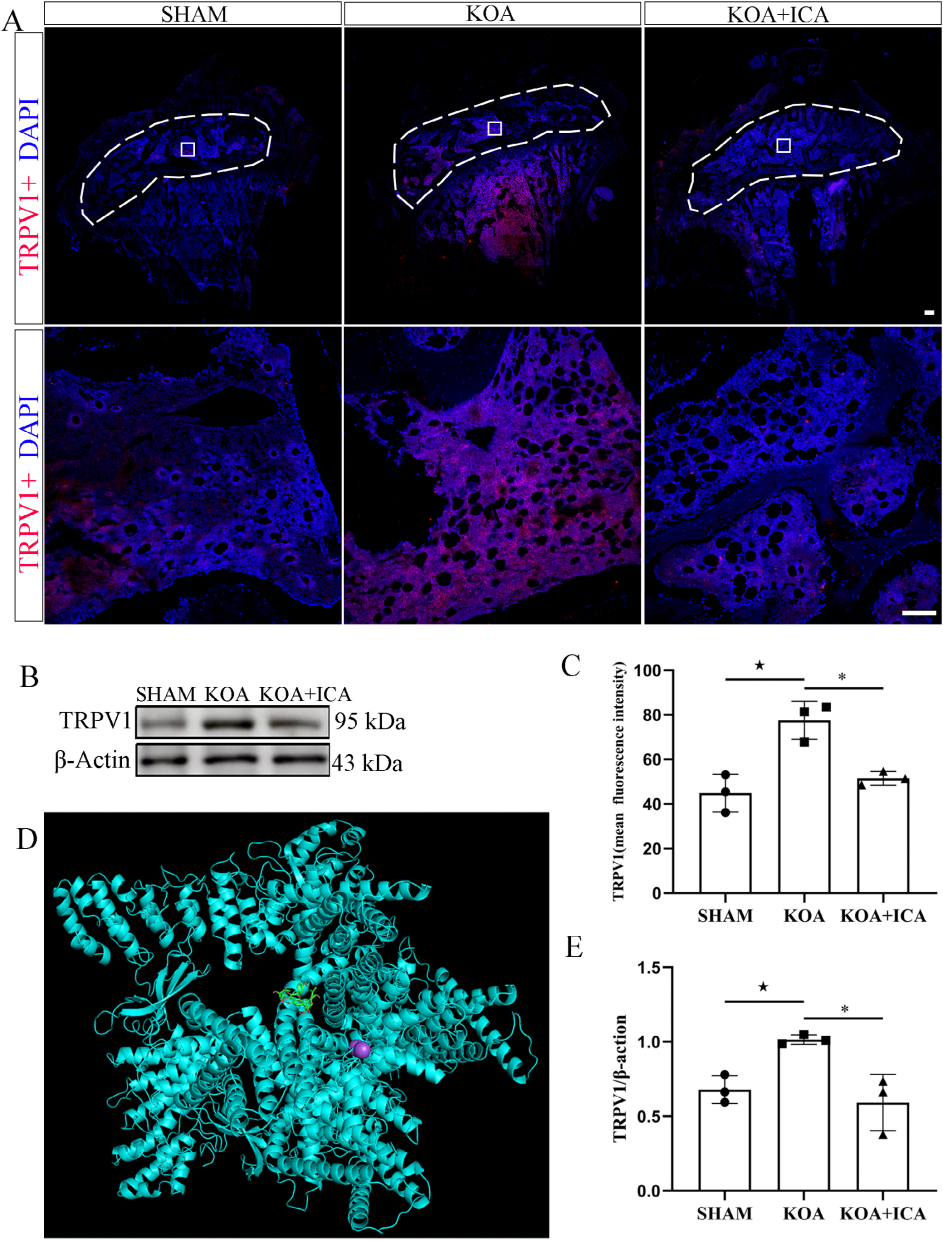
ICA inhibits the expression of TRPV1 in the subchondral bone. ICA fluorescence localisation analysis of TRPV1, local amplification (A) and quantitative analysis of the fluorescence intensity (C) are shown. The white dotted line in A represents the subchondral bone, and the rectangle shows a partially enlarged area (scale bar = 1000 and 50 μm [A]). Representative images (B) and quantitative analysis of TRPV1 protein (E) in the subchondral bone. Molecular docking results of ICA and TRPV1 (D). ^★^ *p* < 0.05, *p < 0.05. ^★^Comparison between SHAM and KOA, *comparison between KOA and KOA + ICA.

Immunofluorescence analysis of the subchondral bone revealed that CGRP, NF200, TrkA and VEGF were all expressed in the subchondral bone, with higher expression levels in the KOA group than in the sham group. However, the expression levels decreased after the ICA intervention. These findings suggest that ICA can reduce the increased distribution of positive nerve fibres and angiogenesis in K1OA (Figure 4). In addition, the expression location of TRPV1 on the subchondral bone was verified, and the results of immunofluorescence and protein assays were consistent with the expression changes in nerve fibres (Figure 5A–C,E). Furthermore, to investigate the direct effect of ICA on TRPV1 (Figure 5D), molecular docking was performed. The results revealed a binding energy between TRPV1 and ICA of −6.0 kJ/mol (< −5.0 kJ/mol), indicating a strong interaction between TRPV1 and ICA.

### 3.4 ICA Regulates the Expression of Positive Nerve Fibres in the Subchondral Bone of KOA Rats Through TRPV1

**FIGURE 6:**
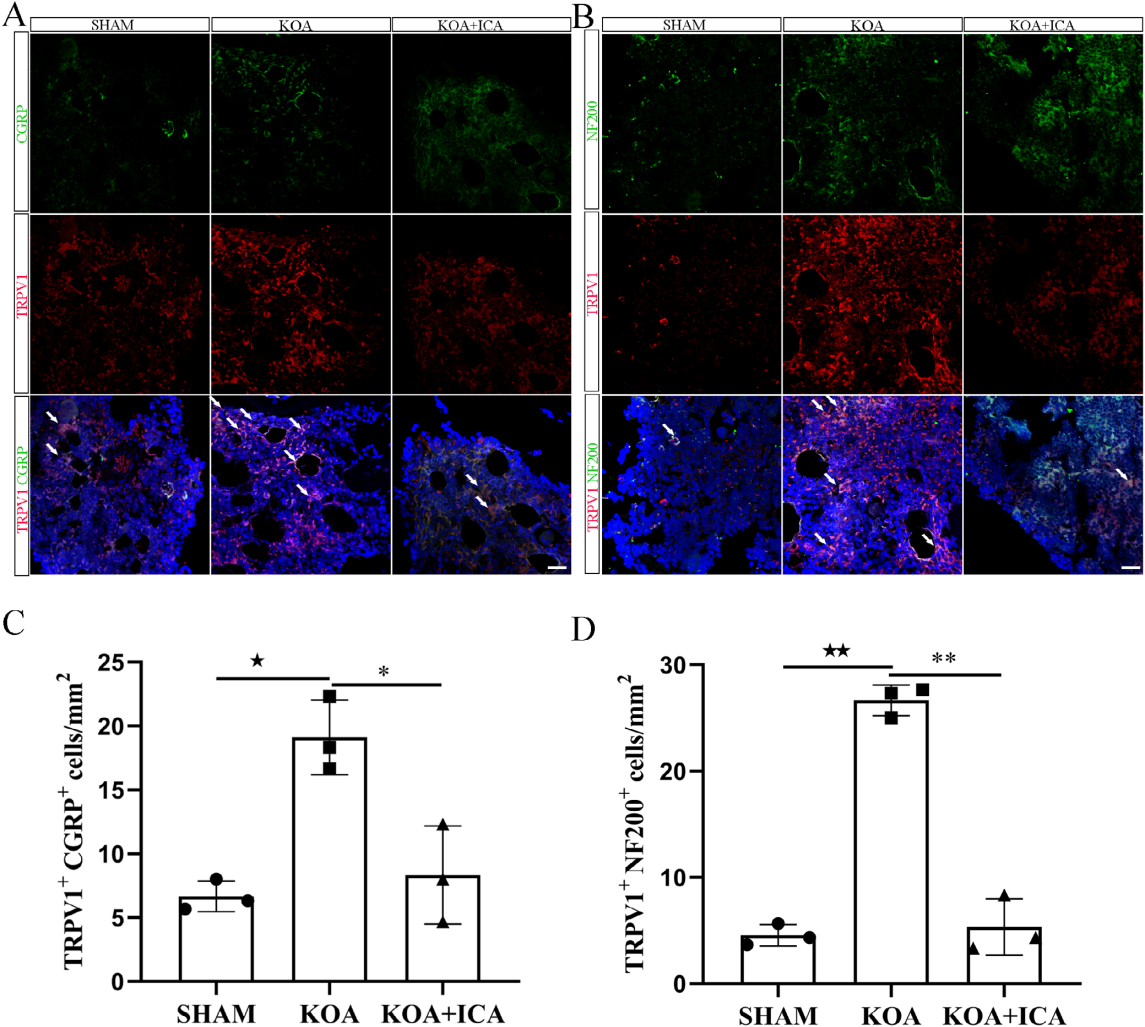
Representative images (A, B) and quantitative analysis (C, D) of colabelled fluorescence for TRPV1, CGRP and NF200 in SHAM, KOA rats and ICA treatment groups (scale bar = 20 μm). The white arrow indicates the colocalisation position. ^★^ *p* < 0.05, ^★★^ *p* < 0.01, *p < 0.05, **p < 0.01. ^★^Comparison between SHAM and KOA, *comparison between KOA and KOA + ICA.

TRPV1 is the main nociceptive transducer involved in pain. Its activation releases neuropeptides such as CGRP, which causes pain. However, blocking TRPV1 can alleviate the pain response induced by mechanical stimulation and reduce the release of CGRP [30]. The colocalisation of TRPV1 and CGRP in the subchondral bone led to a significant increase in the TRPV1 and CGRP numbers in the KOA group compared to the sham group, which was reversed after ICA intervention but did not completely reach the normal level (Figure 6A,B). NF200, which is highly expressed in adult myelinated peripheral axons and is minimally present in cell bodies, marks nerve regeneration [31]. The increased colocalisation of NF200 and TRPV1 in the subchondral bone in the KOA group suggests that activation of the TRPV1 pathway leads to nerve regeneration and pain. The decrease in the NF200 and TRPV1 costandard numbers after ICA intervention indicates that ICA can inhibit nerve regeneration (Figure 6C,D).

### 3.5 ICA Regulates the Inhibitory Effect of Nogo‐A on TRPV1 Channel in the DRG

**FIGURE 7:**
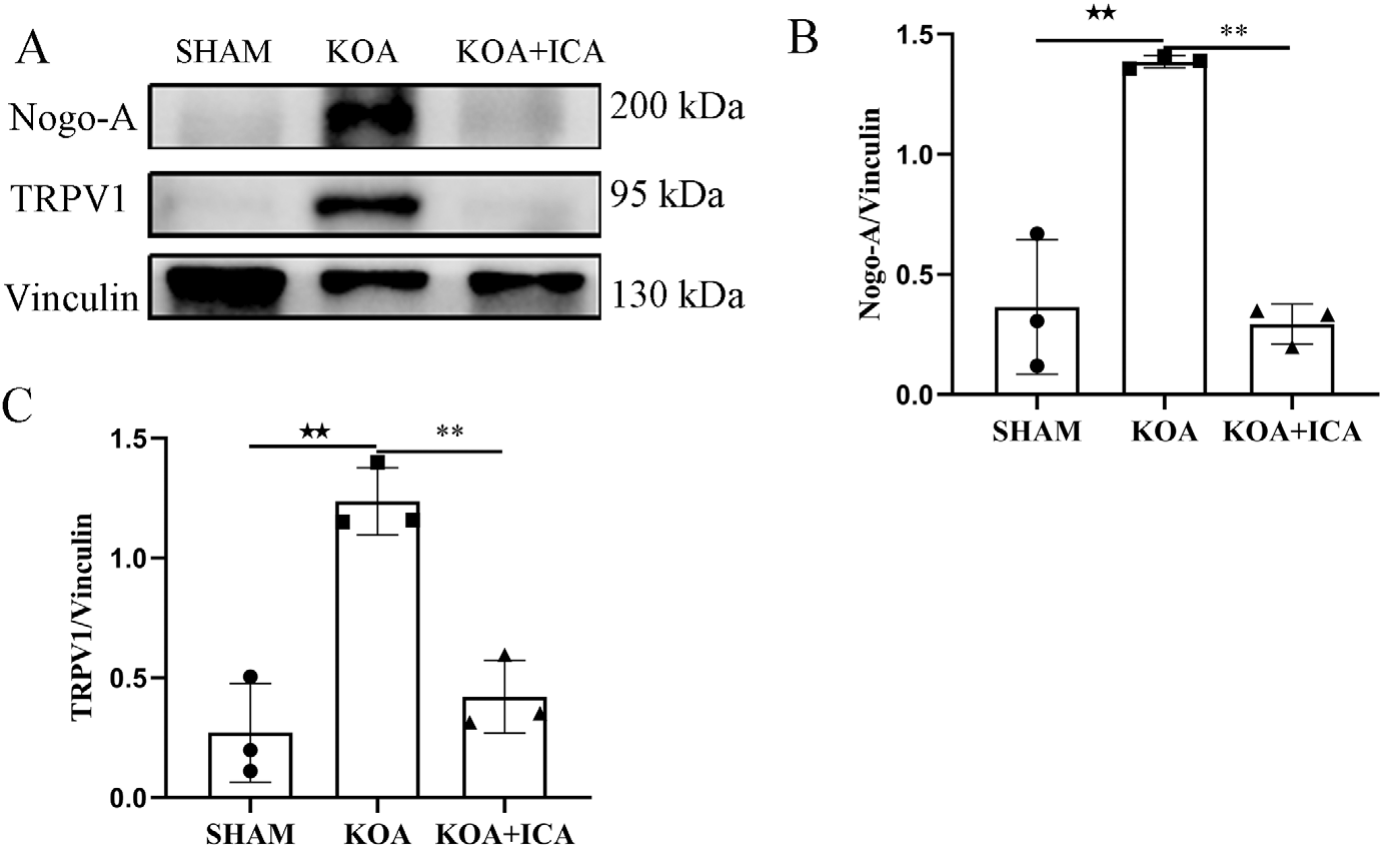
ICA can inhibit the expression of Nogo‐A and TRPV1 in the DRG. Representative images and quantitative analysis of Nogo‐A and TRPV1 in the DRG are shown (A–C). ^★★^ *p* < 0.01, **p < 0.01. ^★^Comparison between SHAM and KOA, *comparison between KOA and KOA + ICA.

Nogo‐A is involved in TRPV1 pathway in the DRG, and its inhibitors can reduce TRPV1 channel activation, resulting in pain relief. In this study, the expression levels of Nogo‐A and TRPV1 proteins in the DRG were significantly higher in KOA group compared to the sham group (*p* < 0.01), as demonstrated by Western blot assays. However, following ICA intervention, the expression levels of both proteins decreased compared to those in the KOA group (*p* < 0.01) (Figure 7A–C). These results indicate that ICA can regulate the expression of Nogo‐A and TRPV1 proteins in the DRG of KOA rats, suggesting that ICA may modulate pain regulation in KOA by affecting TRPV1 through Nogo‐A.

## 4 Discussion

**FIGURE 8:**
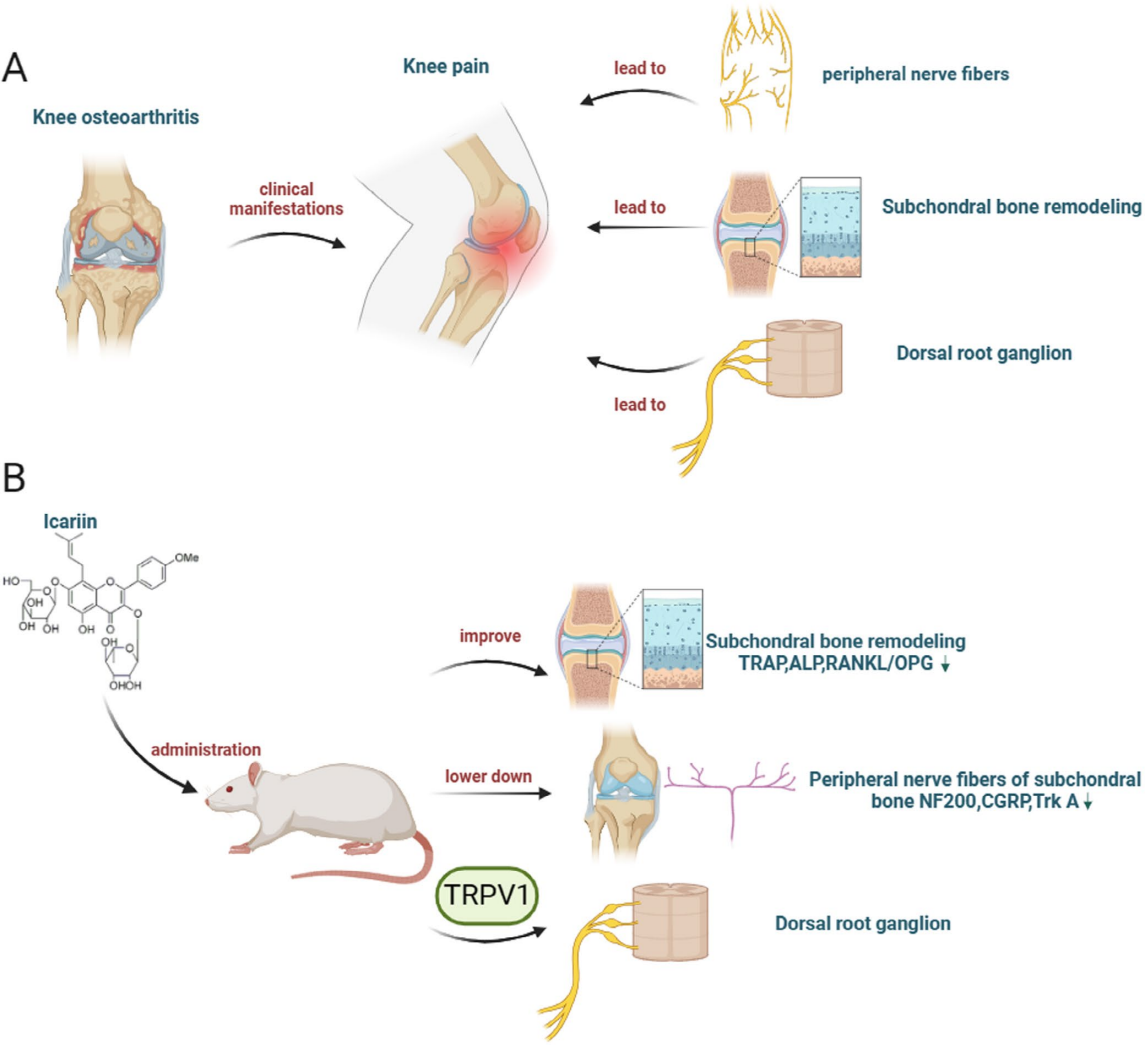
The main causes of KOA pain (A). ICA improves pain‐related mechanism in KOA rats (B).

Subchondral bone lesions significantly contribute to pain in KOA [5], and subchondral bone remodelling plays an important role in advanced KOA [32]. However, the precise mechanism by which ICA relieves KOA‐related pain through its impact on subchondral bone remodelling is not fully understood. In this study, ICA was found to effectively relieve subchondral bone remodelling and reduce KOA‐related pain. At the same time, ICA also reduced the content of peripheral nerve fibres and VEGF in the subchondral bone. In addition, ICA reduced the content of TRPV1 in peripheral nerve fibres as well as the expressions of TRPV1 and Nogo‐A in the DRG. These findings confirm the correlation between ICA, pain relief in KOA and TRPV1 channels in peripheral nerve fibres (Figure 8). However, further follow‐up experiments are required to determine whether pain‐relieving of ICA effects in KOA are directly attributed to subchondral bone remodelling.

### 4.1 Verification of the Therapeutic Effects of ICA

KOA is a chronic bone and joint disease characterised pathologically by the degeneration of articular cartilage and abnormal remodelling of subchondral bone. Chronic pain is a common clinical symptom associated with KOA. Our previous study revealed the potential of ICA for relieving KOA‐related pain. Furthermore, proteomic analysis revealed alterations in pain‐related neuronal proteins and identified relevant brain regions [22]. Gender differences can affect KOA‐related pain. However, there are significant differences between human KOA and animal models. Unlike human KOA patients, the male mice and rats are more susceptible to OA‐like joint degeneration than their female counterparts [33, 34]. In addition, oestrogen and other sex hormones in female rats play a crucial role in the pathogenesis of osteoarthritis and influence KOA‐related pain [35]. Hence, most studies have utilised the male mice or rat model to explore the mechanism and therapeutic effect on KOA in order to eliminate the effects of oestrogen [36, 37].

In this study, we found that the surgical procedure used to establish the KOA model significantly exacerbated the pathological manifestations in these animals. The cartilage layer thickness was reduced, and the structure of the trabecular bone was distorted. However, ICA intervetion could significantly restore these changes. These findings confirmed the successful establishment of a rat model of KOA via surgery. Importantly, we also confirmed the analgesic effect of ICA in a rat model of KOA. In this study, we determined the PWT and PWL to confirm that ICA can relieve KOA‐related pain, which is consistent with our previous findings [22].

Neuropeptides, such as CGRP, SP and TH, play an important role in the transmission and regulation of KOA‐related pain. SP can activate neurons and inflammatory cells, leading to pain perception and exacerbation of the inflammatory response [28]. CGRP can synergise the proinflammatory and pain‐inducing effects of SP. Additionally, CGRP can promote pain sensation and inflammatory responses by activating neurons and inflammatory cells [38]. TH, as a key enzyme in the synthesis of the neurotransmitter norepinephrine, is secreted by sympathetic nerves and participates in bone metabolism and inflammation. It is highly expressed in KOA cartilage, synovium and subchondral bone as a rate‐limiting enzyme in catecholamine biosynthesis expressed by sympathetic nerve fibres. Changes in mechanical stress can lead to the sprouting of TH^+^ nerve fibres in the subchondral bone [39]. Therefore, SP, CGRP and TH are closely associated with KOA‐related pain. In this study, we measured the levels of SP, CGRP and TH in the serum of rats in each group and found that ICA can modulate their expression. Furthermore, ICA can also influence changes in the pain‐related factor NGF in the subchondral bone.

Subchondral bone is highly sensitive to noxious stimuli and is closely associated with KOA‐related pain [40]. Imaging findings have demonstrated a close relationship between chronic joint pain and pathological changes in the subchondral bone structure [41]. In this study, we confirmed the effectiveness of ICA in reducing subchondral bone remodelling.

The progression of KOA is determined by the activity of osteoblasts and osteoclasts. During bone formation, osteoblasts can further differentiate into osteocytes, which are embedded in the mineralised matrix and have distinct morphology and function. Osteoclasts, conversely, are large, multinucleated cells that resorb bone and contribute to bone development and remodelling [42, 43].

The RANKL/OPG ratio serves as an indicator of bone health, reflecting the balance between bone formation and resorption. In KOA, the pathophysiological changes associated with arthritis lead to alterations in the RANKL/OPG ratio, which promote bone resorption and remodelling [44]. Excessive osteoclast generation and enhanced bone resorption activity accelerate the process of bone resorption and remodelling, increasing the RANKL/OPG ratio and ultimately leading to joint destruction and pain [45, 46]. The analyses of subchondral bone morphology and the expression of subchondral bone remodelling–related proteins revealed that the expression of these proteins was upregulated in the KOA group and downregulated in the ICA group. These findings suggest that KOA enhances bone remodelling, while ICA may attenuate these changes.

### 4.2 ICA Improves Knee Osteoarthritis–Related Pain by Improving the Expression of Positive Fibres

The mechanism of pain in KOA is complex. While cartilage itself is not innervated, nociceptors are present in the subchondral bone, synovium and surrounding joint tissues. Peripheral and central sensitisations, which are characteristic of chronic pain conditions, are strongly associated with the severity of KOA‐related pain. Peripheral sensitisation, in particular, is influenced by changes in the subchondral bone. In the KOA group, the expression levels of certain molecules, such as CGRP, NF200, TrkA and VEGF, which are related to angiogenesis, were all increased. However, their expression levels significantly decreased after the ICA intervention.

Neurons contributing to chronic pain include small myelinated neurons (Aδ fibres) and unmyelinated neurons (C fibres). These neurons can be further categorised into peptidergic neurons, which are positive for CGRP, and nonpeptidergic neurons, which are positive for isolectin B4 [9]. Peptidergic neurons are regulated by NGF, express a high affinity for TrkA and are highly sensitive to inflammation. In contrast, sensory neurons with larger or medium‐sized diameters and myelinated or thinly myelinated axons (A fibres) are labelled with NF200. NF200 serves as a marker of myelinated fibres and helps distinguish them from unmyelinated fibres [8]. Pain‐related CGRP plays an important role in pain conduction and regulation in OA [47, 48]. CGRP is derived from sensory nerve fibres and is highly expressed in the articular cartilage and synovial fluid of patients with OA. Its activation can exacerbate pain sensations and inflammatory responses by activating neurons and inflammatory cells. Additionally, it may be involved in regulating osteoclast differentiation during bone remodelling and inhibiting bone resorption by activating the RANKL/OPG signalling pathways [49].

VEGF plays a key role in promoting angiogenesis and repair and is associated with pain in OA. The angiogenesis and innervation mediated by VEGF contribute to subchondral bone remodelling during OA progression. VEGF can promote bone resorption and destruction by recruiting and activating osteoclasts and can directly activate sensory neurons. In OA, VEGF expression is increased in articular tissues, particularly subchondral bone tissues. Evidence suggests that angiogenesis inhibitors can reduce pain‐related behaviours in rats with OA. Furthermore, these inhibitors reduce angiogenesis at the affected joint site in rats with KOA, as well as pain‐related behaviours, suggesting a potential role of angiogenesis in KOA‐related pain [50, 51].

TRPV1 is enriched in the small‐diameter cell bodies of nociceptive nerve fibres innervating joints and joint capsules. It is upregulated in sensory afferent fibres innervating OA joints and is associated with pain in animal models of OA and in human patients with OA. In addition, TRPV1 is involved in the formation of osteoblasts and osteoclasts in the subchondral bone and can contribute to bone pain. Blocking TRPV1 can relieve OA‐related pain. The efficacy of axon terminal ablation therapy targeting TRPV1 channels in neuropathic pain has been clearly demonstrated [12]. In this study, TRPV1 expression, which was found to be highly expressed in the KOA group, was significantly reduced following ICA intervention.

These findings suggest that there are multiple nerve fibres involved in KOA‐related pain. Positive fibres can exacerbate inflammation and bone remodelling through TRPV1, leading to peripheral and central sensitisation and the development of pain. However, ICA treatment has the potential to reverse these changes.

### 4.3 ICA Improves Knee Osteoarthritis–Related Pain by Inhibiting the Nogo‐A/TRPV1 Pathway in the Dorsal Root Ganglion

Our previous research indicates that ICA effectively relieves pain through neuromodulation, and proteomic analysis suggests a close relationship with the Nogo protein. Existing evidence suggests that Nogo‐A can inhibit TRPV1 channel activation in the DRG, resulting in pain relief. However, the impact of ICA on Nogo‐A/TRPV1 in the DRG has not been investigated. In this study, we found that ICA can inhibit the expression and activation of Nogo‐A and TRPV1 in the DRG. These findings provide new insights into the potential of ICA for relieving OA‐related pain.

Peripheral nerve fibres innervating the subchondral bone can be traced back to the DRG. Modulation of the integration network between the DRG, glial cells, neurons and immune cells in the CNS has been linked to arthritic pain [40]. In addition to the cartilage, DRG nociceptors are also found in other areas of the joint, including the joint capsule, synovium, fat pad, ligaments, periosteum and subchondral bone.

TRPV1 is not only widely distributed in the peripheral and central nervous systems but can also be freely transported to peripheral nerve endings and distributed in the periphery [52]. It is also widely distributed in sensory nerve fibres, particularly in nonmyelinated C‐type fibres capable of conducting pain and temperature sensation and partially myelinated Aδ fibres capable of conducting pain and cold sensation [53]. The involvement of TRPV1 in OA‐related pain has been demonstrated. TRPV1 receptors, which are located at the peripheral and central ends of the DRG, shift from small‐diameter to medium‐diameter cells in the presence of peripheral inflammation. This transition leads to peripheral thermal and mechanical hyperalgesia. In addition, TRPV1 receptors increase the release of transmitters in the spinal dorsal horn, leading to long‐term enhancement of synaptic transmission in the spinal cord and central sensitisation of the spinal dorsal horn [54].

The Nogo‐A protein not only inhibits the growth and regeneration of neuronal axons but also plays an important role in pain transmission and regulation. The expression level of the Nogo‐A protein is increased in pathological pain conditions, and using its antagonist can alleviate the severity of pathological pain [55]. In addition, Nogo‐A protein and its receptor NgR1 are expressed in pain‐related neurons and glial cells, contributing to pain conduction and regulation in both the central and peripheral nervous systems. Nogo‐A protein is also involved in the initiation and maintenance of pain through its interaction with neurotransmitters, as well as the formation and regulation of axons and synapses. Furthermore, Nogo‐A inhibitors reduce TRPV1 channel activation in the DRG and relieve pain through various mechanisms. Disrupting Nogo‐A signalling reduces LIM domain kinase/cofilin phosphorylation and actin polymerisation, thereby inhibiting TRPV1 channel function and expression in DRG neurons, while Nogo‐A itself maintains TRPV1 function in the DRG [17]. In rats with complete Freund's adjuvant‐induced arthritis, intrathecal injection of a Nogo‐A receptor antagonist has been shown to reduce the TRPV1 content in the DRG and effectively relieve chronic pain [24]. In this study, we found that the expression of Nogo‐A and TRPV1 proteins in the DRG and their fluorescence colocalisation were higher in the KOA group. However, treatment with ICA reversed this trend. These findings suggest that the Nogo‐A/TRPV1 pathway is involved in the transmission of KOA‐related pain to the DRG, and ICA treatment can inhibit the transmission of these pain signals.

## 5 Conclusion

In this study, we verified the therapeutic effect of ICA on a rat model of KOA and investigated the neural mechanism of pain. The observed morphological changes and phenotypic alterations in pain behaviours in KOA rats provided evidence for the effectiveness of ICA treatment. We further investigated the peripheral neural mechanism of ICA treatment for KOA by examining the expression of positive nerve fibres in the subchondral bone and the fluorescence colocalisation of TRPV1. Interestingly, we found that the expression changes of the TRPV1 pathway in the DRG were similar to those in the subchondral bone. These findings indicate the involvement of peripheral nerves in the pain‐relieving effects of ICA in KOA. Furthermore, we found a close association between TRPV1 and the peripheral neural mechanism underlying ICA's pain‐relieving effect in KOA. These findings support ICA as a potential new treatment for KOA.

## Author Contributions

**Dezun Ma:** conceptualization (equal), data curation (equal), methodology (equal), supervision (equal), writing – review and editing (equal). **Zaishi Zhu:** conceptualization (equal), data curation (equal), investigation (equal), methodology (equal), writing – original draft (equal). **Xue Tan:** conceptualization (equal), data curation (equal), writing – original draft (equal). **Qing Lin:** formal analysis (equal), validation (equal). **Yanfeng Huang:** formal analysis (equal), validation (equal). **Min Mao:** methodology (equal), visualization (equal). **Zhouping Yi:** methodology (equal), visualization (equal). **Lili Wang:** investigation (equal). **Linglong Liu:** methodology (equal). **Xihai Li:** funding acquisition (equal), supervision (equal), writing – review and editing (equal).

## Ethics Statement

The animal study was reviewed and approved by the Animal Care and Use Committee of the Fujian University of TCM (Approval Number: 2019061).

## Conflicts of Interest

The authors declare no conflicts of interest.

## Supporting information


**Figure S1.** Mechanical pain sensitivity was assessed weekly after modelling using the PWT.

## Data Availability

The data that support the findings of this study are avalable from the corresponding author upon reasonable reguest.
