# Supplementary material for: Validation of Peripheral Neuromodulation Mechanisms of Icariin in Knee Osteoarthritis–Related Chronic Pain
Source: J Cell Mol Med. 2024 Dec 2;28(23):e70223. doi: 10.1111/jcmm.70223 (PMC11611524; doi:10.1111/jcmm.70223)
Supplement: Supplementary file 1 — Figure S1. Mechanical pain sensitivity was assessed weekly after modelling using the PWT. [file JCMM-28-e70223-s001.docx]

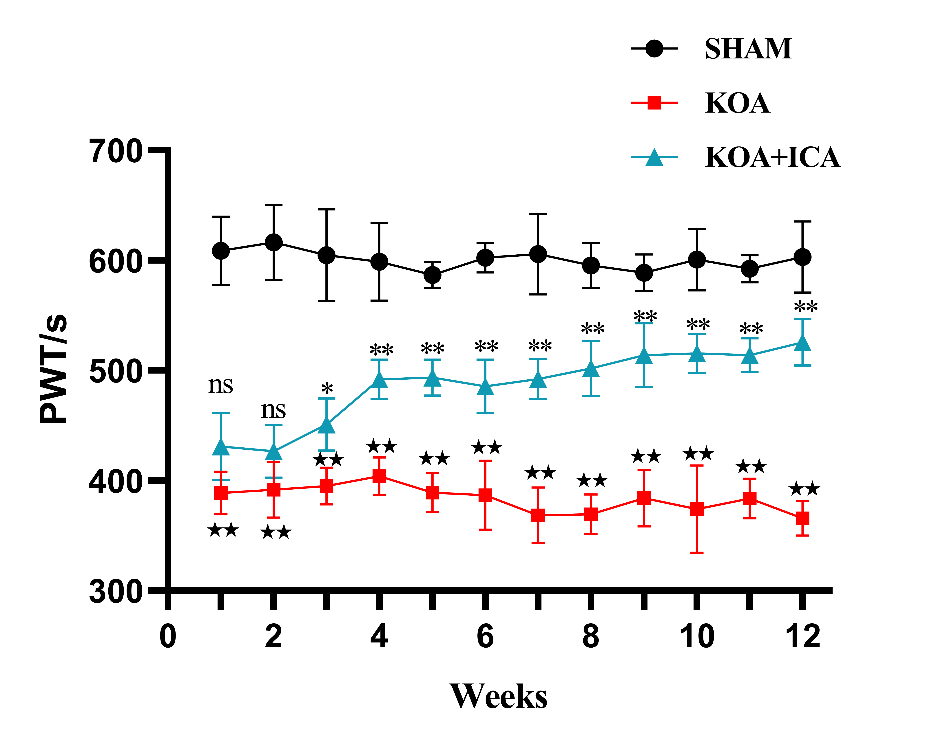


Figure S1 Mechanical pain sensitivity was assessed weekly after modelling using the PWT. ^★★^*p* < 0.01, **p* < 0.05, ***p* < 0.01, ns for no significance. ^★^Comparison between SHAM and KOA, *comparison between KOA and KOA + ICA.
